# Effects of R219K polymorphism of ATP-binding cassette transporter 1 gene on serum lipids ratios induced by a high-carbohydrate and low-fat diet in healthy youth

**DOI:** 10.1186/0717-6287-47-4

**Published:** 2014-03-26

**Authors:** Hui Liu, Jia Lin, Xing Chun Zhu, Yuan Hao Li, Mei Fan, Rong Rong Zhang, Ding Zhi Fang

**Affiliations:** Department of Biochemistry and Molecular Biology, West China School of Preclinical and Forensic Medicine, Sichuan University, Chengdu, 610041 P. R. China

**Keywords:** Apolipoprotein, ATP-binding cassette transporter 1 gene, Gene polymorphism, High-carbohydrate/low-fat diet, Lipid ratios

## Abstract

**Background:**

Diets are the important players in regulating plasma lipid profiles. And the R219K polymorphism at the gene of ATP-binding cassette transporter 1(*ABCA1*) was reported to be associated with the profiles. However, no efforts have been made to investigate the changes of lipid profiles after a high-carbohydrate and low-fat diet in different subjects with different genotypes of this polymorphism. This study was to evaluate the effects of *ABCA1* R219K polymorphism on serum lipid and apolipoprotein (apo) ratios induced by a high-carbohydrate/low-fat (high-CHO) diet. After a washout diet of 54.1% carbohydrate for 7 days, 56 healthy young subjects (22.89 ± 1.80 years old) were given a high-CHO diet of 70.1% carbohydrate for 6 days. Height, weight, waist circumference, hip circumference, glucose (Glu), triglyceride (TG), total cholesterol (TC), high density lipoprotein cholesterol (HDL-C), low density lipoprotein cholesterol (LDL-C), apoA-1 and apoB-100 were measured on the 1^st^, 8^th^ and 14^th^ days of this study. Body mass index (BMI), waist-to-hip ratios (WHR), log(TG/HDL-C), TC/HDL-C, LDL-C/HDL-C and apoA-1/apoB-100 were calculated. *ABCA1* R219K was analyzed by a PCR-RFLP method.

**Results:**

The results indicate that the male subjects of all the genotypes had higher WHR than their female counterparts on the 1^st^, 8^th^ and 14^th^ days of this study. The male K carriers had higher log(TG/HDL-C) and TC/HDL-C than the female carriers on the 1^st^ and 14^th^ days, and higher LDL-C/HDL-C on the 14^th^ day. When compared with that on the 8^th^ day, TC/HDL-C was decreased regardless of the genotypes and genders on the 14^th^ day. Log(TG/HDL-C) was increased in the males with the RR genotype and the female K carriers. Lowered BMI, Glu and LDL-C/HDL-C were found in the male K carriers, but only lowered BMI in the female K carriers and only lowered LDL-C/HDL-C in the females with the RR genotype.

**Conclusions:**

These results suggest that *ABCA1* R219K polymorphism is associated differently in males and females with elevated log(TG/HDL-C) and decreased LDL-C/HDL-C induced by the high-CHO diet.

## Introduction

Epidemiologic studies have shown a strong relationship between cardiovascular disease (CVD) and the changes of serum lipids and apolipoproteins [1–3]. High levels of low density lipoprotein cholesterol (LDL-C) and apolipoprotein (apo) B-100, as well as low levels of high density lipoprotein cholesterol (HDL-C) and apoA-1 are regarded to be the crucial and independent risk factors of CVD [1, 4]. Serum lipids and apolipoproteins are modulated by both environmental and genetic factors [5, 6]. For example, the consumption of different diets, which is considered to be a key environment factor, can lead to diverse changes of serum lipids and apolipoproteins [7, 8]. Evidences show that high carbohydrate diet can lead to hypertriglyceridemia by raising serum triglyceride (TG) and decreasing HDL-C [7, 9]. However, after a high carbohydrate and low fat (high-CHO) diet was introduced, reduction of LDL-C was observed [10]. Meanwhile, investigations have been carried out to evaluate the effects of the high-CHO diet on the profiles of serum lipids and apolipoproteins in different genetic backgrounds [11–13]. However, the mechanisms underlying the changes of serum lipids and apolipoproteins after the high-CHO diet have not been revealed yet. Besides, the ratios of serum lipids and apolipoproteins, including log(TG/HDL-C), TC/HDL-C, LDL-C/HDL-C and apoA-1/apoB-100, are thought to be better in the prediction and evaluation of CVD [14–16]. Changes of serum lipid levels in response to dietary modifications vary greatly among individuals in the general population and are, in part, genetically controlled, potentially by the genes of the key proteins participating in the metabolism of lipids and lipoproteins.

The ATP-binding cassette transporter 1 (ABCA1), a membrane transporter protein, plays a key role in regulating serum HDL-C and apoA-1 metabolism [17, 18]. It can stimulate cholesterol and phospholipid efflux to apoA-1 [18], one of the first stages in the reverse cholesterol transport (RCT) which mediates the cholesterol catabolism from peripheral cells back to the liver. Therefore, it has been considered as a rate limiting step in the production of HDL. The single nucleotide polymorphisms (SNPs) are relatively common in the ABCA1 gene (*ABCA1*) [19–21] and several polymorphic variants affecting the amino acid sequence have recently been published [22, 23]. One of the most common missense polymorphisms in the coding region of the *ABCA1* is R219K polymorphism with an allele frequency of the K allele of 46% in the European population [24]. Several studies have found decreased TG and increased HDL-C levels in the K219 homozygotes [17, 25]. It is believed that the K allele of R219K polymorphism alone is an independent protective factor against CVD. However, little is known about the interactions between the R219K polymorphism of *ABCA1* and the high-CHO diet and their effects on serum lipids and apolipoproteins in health youth.

In this study, we investigated the effects of R219K polymorphism of *ABCA1* on the anthropometric parameters and plasma lipid and apolipoprotein ratios after the high-CHO diet in healthy young Chinese, a population well characterized with diets containing high carbohydrate and low incidence of CVD. To our knowledge, these effects have not been tested before.

## Results

### The frequencies of genotypes and alleles of R219K polymorphism of ABCA1

The fragment contained R219K polymorphism site of *ABCA1* was 177 bp. The digestion of the PCR products of the RR homozygotes generated a single fragment of 177 bp, the KK homozygotes resulted in 107 bp and 70 bp fragments, and the RK heterozygotes presented 177 bp, 107 bp and 70 bp fragments. The genotype and allele frequencies of R219K polymorphism of *ABCA1* in this study population are shown in Table 1. No deviation from the Hardy-Weinberg equilibrium was found in the distribution of genotypes (*χ*^2^ =2.357, *P* =0.125). No statistically significant gender difference for genotype frequencies was observed in this study population.

**Table 1:** **Frequencies of the genotype and allele of** ***ABCA1*** **R219K polymorphism**

			Genotype		Allele frequency	
	***N***	RR [case (%)]	RK [case (%)]	KK [case (%)]	R allele	K allele
**Total**	56	20 (35.7)	22 (39.3)	14 (25.0)	0.55	0.45
**Male**	27	8 (14.3)	11 (19.6)	8 (14.3)	0.50	0.50
**Female**	29	12 (21.4)	11 (19.6)	6 (10.7)	0.60	0.40

### The characteristics of the subjects on the 1^st^ day of this study according to R219K genotypes of ABCA1

Due to the small number of the subjects with the KK genotype, the subjects with the RK genotype and the subjects with the KK genotype were combined and defined as the K allele carriers. As shown in Table 2, the males with the RR genotype had significant higher WHR (*P* < 0.05) than their female counterparts. Meanwhile, the male K allele carriers had significantly higher WHR (*P* < 0.001), log(TG/HDL-C) (*P* < 0.05) and TC/HDL-C (*P* < 0.05) than the female carriers.

**Table 2:** **Anthropometric and biochemical parameters of the subjects on the 1** ^**st**^ **day of this study**

Variables	Males (***n*** = 27)		Females (***n*** = 29)		Total (***n*** = 56)	
	RR	K carriers	RR	K carriers	RR	K carriers
**Age, years**	23.13 ± 2.42	22.89 ± 1.79	23.00 ± 1.21	22.71 ± 1.96	23.05 ± 1.73	22.81 ± 1.85
**BMI, kg/m** ^**2**^	20.87 ± 3.90	22.28 ± 4.25	20.07 ± 2.30	20.42 ± 2.82	20.39 ± 2.97	21.40 ± 3.72
**WHR**	0.89 ± 0.05	0.89 ± 0.06**	0.83 ± 0.04	0.82 ± 0.05	0.85 ± 0.05	0.86 ± 0.06
**Glu, mg/dl**	4.01 ± 0.74	4.00 ± 0.44	4.03 ± 0.57	4.00 ± 0.54	4.02 ± 0.62	4.00 ± 0.48
**log(TG/HDL-C)**	0.12 ± 0.30	0.14 ± 0.29*	-0.03 ± 0.19	-0.08 ± 0.20	0.03 ± 0.24	0.04 ± 0.27
**TC/HDL-C**	2.60 ± 0.87	2.58 ± 0.60*	2.24 ± 0.43	2.19 ± 0.37	2.39 ± 0.64	2.39 ± 0.53
**LDL-C/HDL-C**	1.28 ± 0.73	1.09 ± 0.97	1.02 ± 0.41	1.05 ± 0.44	1.12 ± 0.56	1.07 ± 0.75
**apoA-1/apoB-100**	3.82 ± 1.75	3.09 ± 1.08	3.20 ± 0.92	3.33 ± 0.81	3.45 ± 1.31	3.22 ± 0.94

*P < 0.05, **P < 0.001, compared with the females with the same genotype.

### Effects of the high-CHO diet on anthropometric parameters and plasma lipid ratios of the subjects

As shown in Table 3, there were no significant differences of anthropometric parameters, glucose, lipid ratios and apoA-1/apoB-100 between the subjects with the RR genotype and the K allele carriers on the 8^th^ or 14^th^ day of this study in the total subjects or in the males and females separately. When compared between gender groups, the males with the RR genotype had higher WHR than their female counterparts on the 8^th^ (*P* < 0.05) and 14^th^ (*P* < 0.01) days. Meanwhile, the male K allele carriers had significantly higher WHR (*P* < 0.001), log(TG/HDL-C) (*P* < 0.01), TC/HDL-C (*P* < 0.05) and LDL-C/HDL-C (*P* < 0.05) than the female carriers on the 8^th^ day. However, these differences between genders were diminished and only higher WHR was observed in the male K allele carriers (*P* < 0.001) than in the females on the 14^th^ day. When compared with that on the 8^th^ day, TC/HDL-C (*P* < 0.01) experienced a significant decrease on the 14^th^ day, regardless of the genotypes and genders. The males with the RR genotype experienced a significant increase of log(TG/HDL-C) (*P* < 0.05), while the K allele carriers had lower BMI (*P* < 0.05), glucose (*P* < 0.05) and LDL-C/HDL-C (*P* < 0.001) levels on the 14^th^ day. LDL-C/HDL-C (*P* < 0.01) decreased significantly in the female RR homozygotes. Decreased BMI (*P* < 0.05) and increased log(TG/HDL-C) (*P* < 0.05) were observed in the female K allele carriers.

**Table 3:** **Anthropometric and biochemical parameters of the subjects on the 8** ^**th**^ **and 14** ^**th**^ **days of this study**

Variables	Males (***n*** = 27)		Females (***n*** = 29)		Total (***n*** = 56)	
	RR	K carriers	RR	K carriers	RR	K carriers
**Age, years**	23.13 ± 2.42	22.89 ± 1.79	23.00 ± 1.21	22.71 ± 1.96	23.05 ± 1.73	22.81 ± 1.85
**BMI, kg/m** ^**2**^						
**8** ^**th**^ **day**	20.74 ± 3.94	22.13 ± 4.23	19.90 ± 2.39	20.30 ± 2.71	20.23 ± 3.03	21.27 ± 3.67
**14** ^**th**^ **day**	20.60 ± 3.98	22.03 ± 4.19*	19.87 ± 2.43	20.15 ± 2.64*	20.16 ± 3.07	21.14 ± 3.62**
**Waist-to-hip ratio**						
**8** ^**th**^ **day**	0.89 ± 0.09^#^	0.91 ± 0.04^###^	0.82 ± 0.06	0.83 ± 0.03	0.84 ± 0.06	0.87 ± 0.05
**14** ^**th**^ **day**	0.89 ± 0.05^##^	0.91 ± 0.05^###^	0.83 ± 0.04	0.83 ± 0.03	0.86 ± 0.05	0.87 ± 0.06
**Glu, mg/dl**						
**8** ^**th**^ **day**	4.27 ± 0.42	4.61 ± 0.52	4.51 ± 0.51	4.33 ± 0.54	4.41 ± 0.48	4.48 ± 0.54
**14** ^**th**^ **day**	4.41 ± 0.46	4.32 ± 0.32*	4.34 ± 0.24	4.28 ± 0.49	4.37 ± 0.33	4.30 ± 0.40
**log(TG/HDL-C)**						
**8** ^**th**^ **day**	0.10 ± 0.29	0.21 ± 0.19^##^	0.04 ± 0.16	0.03 ± 0.15	0.06 ± 0.22	0.12 ± 0.19
**14** ^**th**^ **day**	0.19 ± 0.29*	0.17 ± 0.20	0.09 ± 0.16	0.09 ± 0.16*	0.13 ± 0.22**	0.14 ± 0.18
**TC/HDL-C**						
**8** ^**th**^ **day**	2.84 ± 1.10	3.14 ± 0.78^#^	2.76 ± 0.38	2.63 ± 0.40	2.79 ± 0.73	2.90 ± 0.67
**14** ^**th**^ **day**	2.11 ± 0.74**	2.29 ± 0.56***	2.17 ± 0.37***	2.12 ± 0.31**	2.15 ± 0.53***	2.21 ± 0.46***
**LDL-C/HDL-C**						
**8** ^**th**^ **day**	1.21 ± 0.70	1.48 ± 0.64^#^	1.37 ± 0.33	1.12 ± 0.34	1.24 ± 0.49	1.31 ± 0.54
**14** ^**th**^ **day**	0.94 ± 0.25	1.08 ± 0.27***	1.01 ± 0.18**	1.04 ± 0.19	0.98 ± 0.21**	1.06 ± 0.23***
**apoA-1/apoB100**						
**8** ^**th**^ **day**	4.10 ± 2.01	3.00 ± 1.15	3.49 ± 0.92	3.43 ± 0.83	3.73 ± 1.44	3.20 ± 1.02
**14** ^**th**^ **day**	4.33 ± 2.02	3.12 ± 1.23	3.64 ± 0.96	3.46 ± 0.99	3.91 ± 1.47*	3.28 ± 1.12

*P < 0.05, **P < 0.01, ***P < 0.001, compared with that on the 8^th^ day of this study in the subjects with the same genotype (paired *t*-test).

*#P* < 0.05, *##P* < 0.01, *###P* < 0.001 compared with that of the female subjects with the same genotype (ANOVA).

## Discussion

It is important to note that although no significant differences of anthropometric and biochemical parameters were found at baseline between genotype subgroups in our study. The responses of anthropometric parameters, glucose, lipid ratios and apoA-1/apoB-100 differed significantly in the subjects with different genotypes to the high-CHO diets. The mechanism underlying is not clear. However, previous studies have shown that ABCA1 plays an important role in regulating plasma HDL-C and apoA-1 metabolism [17, 18]. R219K polymorphism is located on the first intracellular region of ABCA1 protein, where glycosylation sites can be found. Evidences also show that this region is strongly associated with the activity of ABCA1 protein [19]. Evans’s work revealed that the allele K219 of *ABCA1* displayed protective effects against CVD [26]. Meanwhile, this allele of *ABCA1* has been indicated to be related to lower serum TG as it could enhance the exchange of cholesterol esters and TG induced by cholesterol esters transfer protein (CETP), which leads to the hydrolysis of TG and HDL by hepatic lipases [24, 26]. On the other hand, the metabolism of intracellular cholesterol and phospholipid can be changed by the increased activity of ABCA1. As a result, the synthesis of fatty acids is instructed from TG to phospholipids [27]. Taken together, the evidences suggest that the metabolism of TG in the K allele carriers is stronger than that in the subjects with the RR genotype. In the present study, due to the reduction of lipid consumption and greater intake of carbohydrate, the absorbed carbohydrate is converted to TG and stored in adipose tissue or other organs after the high-CHO diet. As the higher metabolism of TG in the K allele carriers, no significant hypertriglyceridemia phenomena were observed. However, the males with the K allele might be more susceptible than the female counterparts to the favorable effect of R219K polymorphism on serum TG (Table 3), which may be induced by the steroids or other sex hormones [28]. Besides, TG is mainly exported by the Class B scavenger receptor type I (SR-BI) pathway in females, and the capacity of TG outflow via ABCA1 pathway in females is weaker than that in males [29]. Notwithstanding the anti-atherosclerosis effects of estrogen is mainly activated by ABCA1 pathway [30], no significant differences of the ratios associated with HDL-C were found between the males and the females before or after the high-CHO diet. However, the lipid metabolism is a multi-gene regulated result, and there could be interactions between polymorphisms of one gene and other genes. In this study, the high carbohydrate intake was found to be positively associated with log(TG/HDL-C) in the male subjects with the RR genotype and the female K allele carriers, but negatively associated with LDL-C/HDL-C in the male K allele carriers and the female subjects with the RR genotype, respectively.

During the 6 days of diet intervention in this study, it is conceivable that the changes of the ratios of lipids and apolipoproteins were only associated with genetic variations involved in the metabolism of carbohydrates and lipoproteins during our study period. To our knowledge, this is the first attempt to investigate the effects of the high-CHO diet on serum lipid ratios and apoA-1/apoB-100 in young subjects with different genotypes of R219K polymorphisms of *ABCA1* in a Chinese young population well characterized with a diet of higher carbohydrate and lower fat and a lower incidence of CVD. In order to evaluate the effects of dietary intervention on lipid ratios and apoA-1/apoB-100 in healthy young subjects with different genotypes, we compared not only the lipid ratios and apoA-1/apoB-100 in different genotype subgroups on the 14^th^ day of this study but also the lipid ratios and apoA-1/apoB-100 on the 8^th^ and 14^th^ days among subjects with the same genotype. Therefore, the differences in changes of carbohydrate and lipid biochemistry profiles upon the high-CHO diet intervention were most likely attributed to the specific genetic backgrounds of individuals and the high-CHO diet. Meanwhile, we instructed the subjects to eat to their satiation as usual, because the real intake of energy of people is dominated by each one’s satiation and cannot be isoenergetic in real life. Besides, the carbohydrates and lipids responses to the high-CHO diet can also be effected by the variation of energy intake.

Recently, researches have focused on the effects of carbohydrate quality and quantity on CVD risk [9, 31, 32]. A series of studies have shown that increased carbohydrate consumption, especially simple carbohydrate, has been associated with elevated plasma TG and decreased TC, HDL-C and LDL-C [33]. Moreover, unchanged levels of these lipids, or even decreased TG and increased HDL-C and LDL-C, were reported after the high-CHO diet [34]. These findings reveal that the complexity of lipid metabolism induced by the high-CHO diet. However, almost all of the studies were carried out in middle-aged or senior populations.

The limitation of the present study is that it was carried out in a sample of modest size. The effect of the modest sample size should be considered when the results are explained, especially after the sample was subdivided by gender. For example, the result shows that TC/HDL-C significantly decreased for all genders and all genotypes (Table 3). We cannot exclude the possibility that this constant decrease regardless of genders and genotypes was resulted from, at least partially, the modest sample size. More studies are needed to test whether this finding is constant in a larger population of the same ethnicity and ages.

## Conclusion

In conclusion, the high-CHO diet was found to elevate log(TG/HDL-C) in the male subjects with the RR genotype and the female K allele carriers, but decrease LDL-C/HDL-C in the male K allele carriers and the female subjects with the RR genotype. Once confirmed by bigger population and multi-center trials, the findings could provide a new point of view for personalized dietary intervention for the subjects with different genotypes of the R219K polymorphism of *ABCA1* to reduce the risks of CVD, especially in a country with a quarter of the world's population.

## Methods

### Subjects

Volunteers were recruited at West China Medical Center, Sichuan University. Participants (56 in total, 27 males and 29 females) who met the exclusion and inclusion criteria summarized in a previous publication were selected [35]. They were apparently healthy as indicated by the medical questionnaires and physical and laboratory examinations. All of the subjects were Chinese Han people with the understanding and the written consents. The study protocol was approved by the Human Research Ethics Committee of Sichuan University.

### Diets

A washout diet for 7 days followed by a high-CHO diet for 6 days was introduced to all the participates. The components of the washout and the high-CHO diets have been described before [36]. Briefly, 30.1% of the total energy of the washout diet was derived from fat, 54.1% from carbohydrates, and 15.8% from proteins. 13.8% of the total energy of the high-CHO diet was derived from fat, 70.1% from carbohydrates, and 16.1% from proteins. The meals were prepared from local foods by Department of Nutrition, West China Hospital, Sichuan University. The volunteers always ate together in a group at the student canteen on the campus of Sichuan University. The subjects were asked to eat to their satiation as usual at each meal and not to eat any other food or drink except water. Daily checklists were used to evaluate the compliance of all the subjects to the study design, including what they ate every day. Only the data of the volunteers who had good compliance were included in the final analyses.

### Blood sampling and laboratory examinations

On the mornings of the 1^st^, 8^th^ and 14^th^ days of this study, twelve hour-fasting venous blood samples were collected. Height, weight, waist circumference, hip circumference were measured in duplicate, while serum TG, TC, HDL-C, LDL-C, apoA-1, apoB-100 and glucose were measured three times by regular methods in the laboratory as described before [35]. Body mass index (BMI) was defined as weight (kg)/[height (m)]^2^, while waist-to-hip ratio (WHR) as waist circumference (m)/hip circumference (m). TG/HDL-C, log(TG/HDL-C), TC/HDL-C, LDL-C/HDL-C and apoA-1/apoB-100 were calculated.

### DNA extraction and genotyping

The fasting blood collection was described above. The DNAout kit (Tianze, Mianyang, China) was applied to extract the genomic DNA from white blood cells. The primers for the amplification of the DNA fragments containing the R219K polymorphism of *ABCA1* by polymerase chain reactions (PCR) were designed according to Clee [24]. The forward sequence of the primer was 5’- GTATTTTTGCAAGGCTACCAGTTACATTTGACAA -3’ and the reverse sequence of it was 5’- GATTGGCTTCAGGATGTCCATGTTGGAA -3’. The cycling conditions were 96°C for 10 min followed by 35 cycles of 96°C for 30 s, 60°C for 45 s, and 72°C for 30 s and a final extension at 72°C for 10 min. The genotype of R219K polymorphism of *ABCA1* was analyzed by *Xag*I enzyme digestion. 3 μL of PCR product was digested at 37°C overnight with 1.5 μL of *Xag*I enzyme (Fermentas, USA) in final volume of 20 μL. The digested fragments were identified on gel electrophoresis of 2.5% agarose.

### Statistical analysis

Data were described as mean ± SD unless described. Normality was tested using the Shapiro-Wilk test. Due to the positively skewed distribution of TG, a log power transformation was applied and it was expressed as log(TG/HDL-C). Hardy-Weinberg Equilibrium was applied to evaluate the population genotype and allele distribution. One-way analysis of variance (ANOVA) was used to compare the variables of the subjects with different genotypes or the subjects of different gender. Paired *t* tests were used to analyze the differences between the values at the 8^th^ and 14^th^ days of this study. Statistical significance was defined as *P* ≤ 0.05.
